# Cajaninstilbene Acid Relaxes Rat Renal Arteries: Roles of Ca^2+^ Antagonism and Protein Kinase C-Dependent Mechanism

**DOI:** 10.1371/journal.pone.0047030

**Published:** 2012-10-09

**Authors:** Dong-Mei Zhang, Yong Li, Wai San Cheang, Chi Wai Lau, Shun-Ming Lin, Qian-Lan Zhang, Nan Yao, Ying Wang, Xin Wu, Yu Huang, Wen-Cai Ye

**Affiliations:** 1 Institute of Traditional Chinese Medicine and Natural Products, College of Pharmacy, Jinan University, Guangzhou, China; 2 Guangdong Province Key Laboratory of Pharmacodynamic Constituents of Traditional Chinese Medicine and New Drugs Research, Jinan University, Guangzhou, China; 3 Institute of Vascular Medicine, Li Ka Shing Institute of Health Sciences and School of Biomedical Sciences, Chinese University of Hong Kong, Hong Kong, China; National Institutes of Health, United States of America

## Abstract

Cajaninstilbene acid (CSA) is a major active component present in the leaves of *Cajanus cajan* (L.) Millsp. The present study explores the underlying cellular mechanisms for CSA-induced relaxation in rat renal arteries. Vascular reactivity was examined in arterial rings that were suspended in a Multi Myograph System and the expression of signaling proteins was assessed by Western blotting method. CSA (0.1–10 µM) produced relaxations in rings pre-contracted by phenylephrine, serotonin, 9, 11-dideoxy-9α, 11α-epoxymethanoprostaglandin F_2α_ (U46619), and 60 mM KCl. CSA-induced relaxations did not show difference between genders and were unaffected by endothelium denudation, nor by treatment with N^G^-nitro-L-arginine methyl ester, indomethacin, ICI-182780, tetraethylammonium ion, BaCl_2_, glibenclamide, 4-aminopyridine or propranolol. CSA reduced contraction induced by CaCl_2_ (0.01–5 mM) in Ca^2+^-free 60 mM KCl solution and by 30 nM (−)-Bay K8644 in 15 mM KCl solution. CSA inhibited 60 mM KCl-induced Ca^2+^ influx in smooth muscle of renal arteries. In addition, CSA inhibited contraction evoked by phorbol 12-myristate 13-acetate (PMA, protein kinase C agonist) in Ca^2+^-free Krebs solution. Moreover, CSA reduced the U46619- and PMA-induced phosphorylation of myosin light chain (MLC) at Ser19 and myosin phosphatase target subunit 1 (MYPT1) at Thr853 which was associated with vasoconstriction. CSA also lowered the phosphorylation of protein kinase C (PKCδ) at Thr505. In summary, the present results suggest that CSA relaxes renal arteries *in vitro* via multiple cellular mechanisms involving partial inhibition of calcium entry via nifedipine-sensitive calcium channels, protein kinase C and Rho kinase.

## Introduction

**Figure 1:**
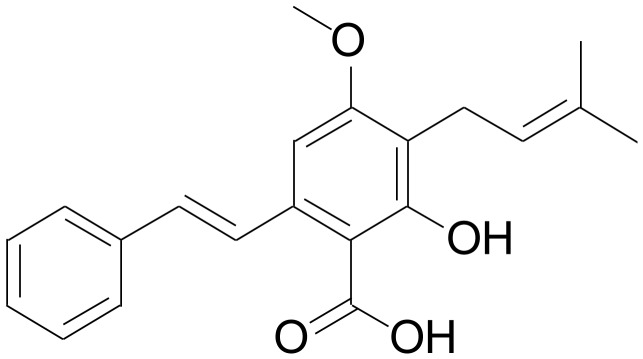
Chemical Structure of cajaninstilbene acid (CSA). The chemical structure of CSA.

Cajaninstilbene acid (CSA, Figure 1), one of the main effective ingredients, is present in the leaves of *Cajanus cajan* (L.) Millsp (pigeon pea) [1] which is commonly used to treat ischemic necrosis of femoral head in traditional Chinese medicine. Recent studies show that the extracts or CSA, possess anti-microbial [2], [3], anti-tumor [4], hepatoprotective [5], [6] and anti-hyperglycemic [7] properties. CSA-containing extracts also protect against amyloid-β 25–35-induced cognitive deficits in mice through increasing the activity of choline acetyl transferase and anti-oxidation [8]. CSA reduces radical and peroxide generation, inhibits xanthine oxidase activity, and protects from DNA damage *in vitro* [9], [10]. The stilbene extracts containing CSA reverse the elevation of the concentration of follicle stimulating hormone and luteinizing hormone and improve femoral morphological structure similar to the effect produced by 17β-estradiol supplementation without affecting the serum 17β-estradiol level and uterine weight in ovariectomized rats, suggesting that CSA may exert a phytoestrogenic activity [11]. In addition, the extract containing 76% CSA markedly lowers levels of serum and hepatic total cholesterol, triglyceride and LDL cholesterol in diet-induced hypercholesterolemic mice, indicating that CSA could be potentially useful for the attenuation of atherosclerosis [12], [13].

The pharmacological activity of CSA in the vascular system is unknown. It is possible that CSA also benefits vascular function. Therefore, the present study was designed to examine the cellular mechanisms for CSA-induced relaxation and roles of signaling molecules involved in the regulation of contractility in rat arteries.

## Results

### The Effect of Cajaninstilbene Acid (CSA) on Agonists-induced Contraction

**Figure 2:**
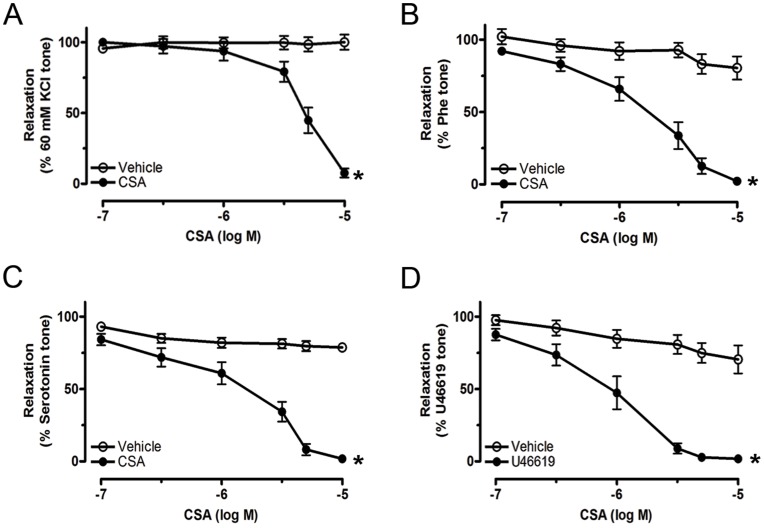
Effect of CSA on agonists-induced contraction. Concentration-response curves for CSA-induced relaxation in rat renal arteries contracted by 60 mM KCl (A), 0.5 µM phenylephrine (Phe) (B), 1 µM Serotonin (C) and 100 nM U46619 (D). Each value represents the mean ± S.E.M of 5–6 independent experiments. *P<0.05 compared with vehicle control.

CSA produced concentration-dependent relaxations to similar degrees in isolated renal arteries pre-contracted with 60 mM KCl, phenylephrine, serotonin and U46619 (Figure 2, Table 1), while the vehicle (DMSO) showed no effect on contraction.

**Table 1:** Responses to CSA in different constrictors.

Constrictor	pD_2_	E_max_ (%)
**60 mM KCl**	5.33±0.03	92.5±7.6^*^
**U46619**	6.13±0.06	98.2±1.4^*^
**Serotonin**	5.96±0.07	98.2±2.6^*^
**Phenylephrine**	5.82±0.06	97.8±2.9^*^

The pD_2_ values and maximum response E_max_ (%) for CSA-induced relaxation in rat renal arteries contracted by different constrictors. Values are means ± S.E.M of n experiments, n = 5−6. *P<0.05 compared with vehicle control.

### Roles of the Endothelium and Estrogen Receptor

**Figure 3:**
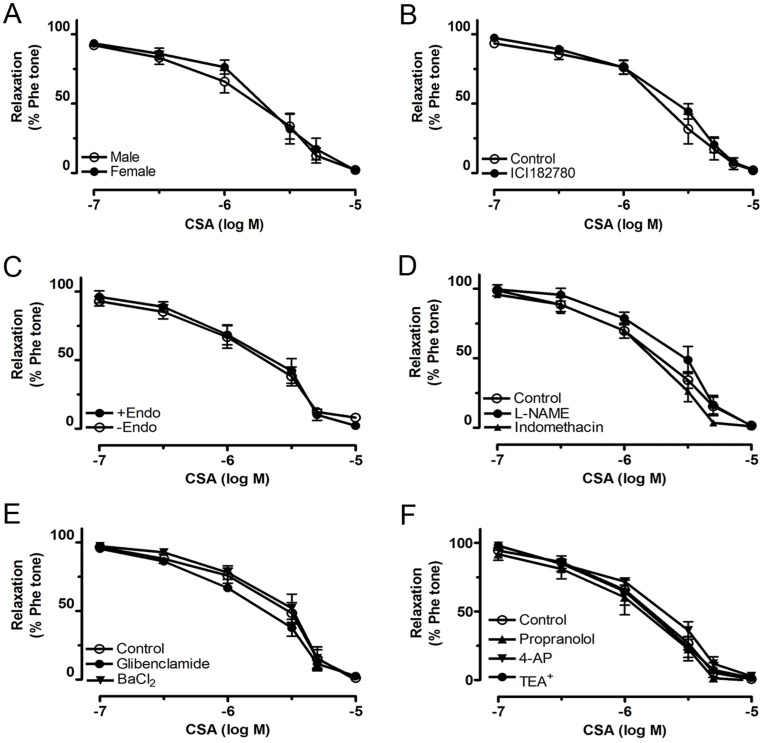
Roles of endothelium and potassium channel blockers on CSA-induced relaxation. Relaxation effect of CSA in renal arteries from male and female rats (A), after 30 min-pretreatment of 10 µM ICI182780 (B), with or without endothelium (C), after 30 min-pretreatment of 100 µM L-NAME or 10 µM indomethacin (D), 10 µM BaCl_2_ or 10 µM glibenclamide (E), and 3 mM TEA^+^, 1 mM 4-AP or 1 µM propranolol (F). Values are means ± S.E.M of 6 experiments.

CSA-induced relaxations showed no gender difference since they were comparable in both male and female rat arteries (Figure 3A). Treatment with ICI-182780 (estrogen receptor antagonist at 10 µM, Figure 3B), L-NAME (nitric oxide synthase inhibitor at 100 µM) and indomethacin (non-selective cyclooxygenase inhibitor at 3 µM) (Figure 3D), or mechanical removal of endothelium (Figure 3C) did not modulate the relaxant effect of CSA.

CSA-induced relaxations were unaffected by 30-min exposure to BaCl_2_ (inwardly rectifying potassium channel blocker at 10 µM), glibenclamide (ATP-sensitive potassium channel blocker at 10 µM), TEA^+^ (calcium-activated potassium channel blocker at 3 mM), 4-AP (voltage-sensitive potassium channel blocker at 1 mM) and propranolol (non-selective β-adrenoceptor antagonist at 1 µM) (Figure 3E and F).

### Roles of Calcium Channel Inhibition in CSA-induced Relaxations

**Figure 4:**
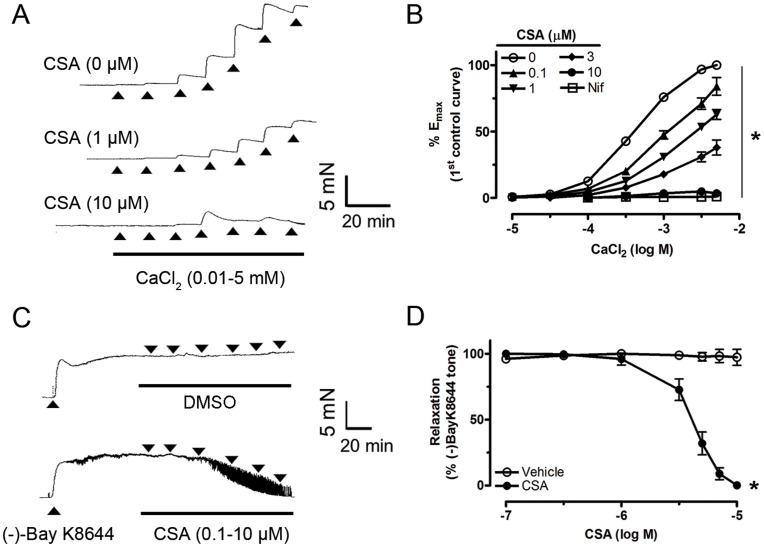
Involvement of calcium channels in CSA-induced relaxation. Representative traces (A) and summarized graph (B) showing CaCl_2_-induced dose-dependent contraction in the absence and presence of different concentration of CSA in rings without endothelium. CSA-induced relaxation in endothelium-denuded rings contracted by 30 nM (−)-Bay K8644 in 15 mM KCl solution (C and D). Values are means ± S.E.M of 6 experiments. *P<0.05 compared with control or vehicle control.

**Figure 5:**
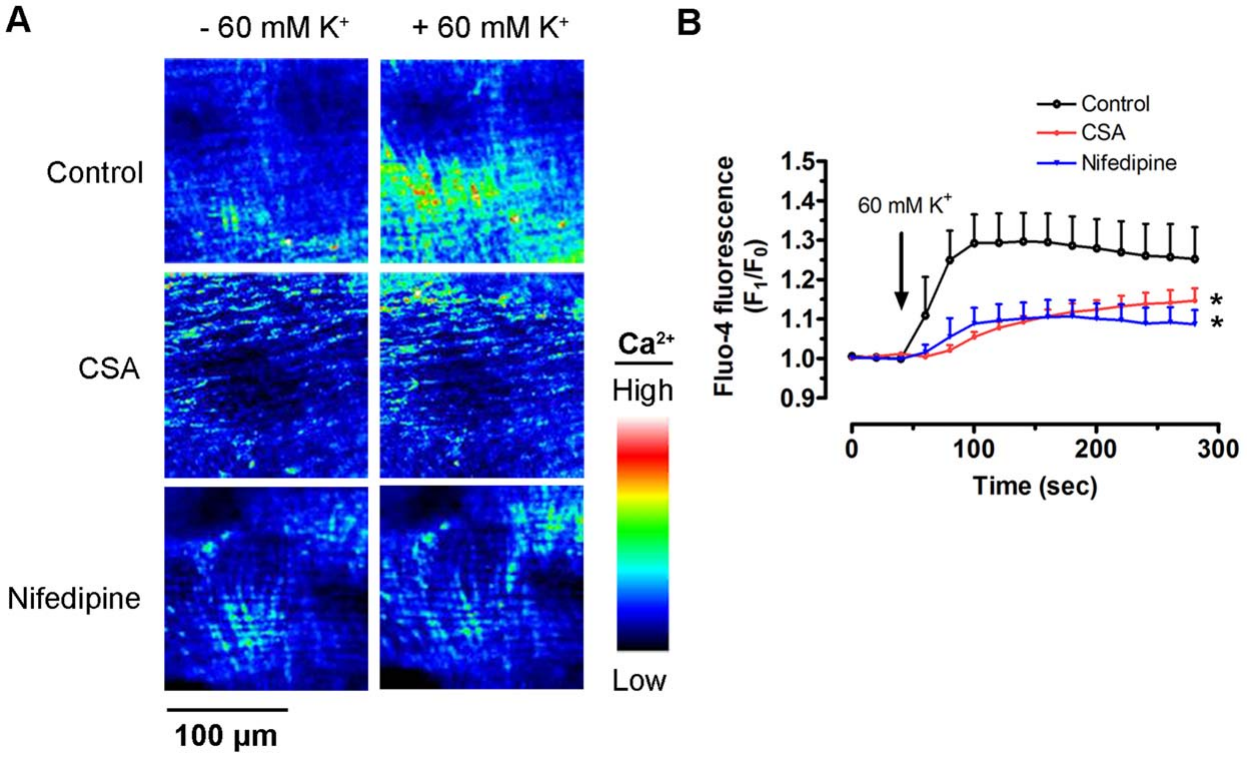
Effect of CSA on Ca^2+^ influx in smooth muscle tissue of renal arteries. Representative images (A) and summarized graph (B) showing the changes of intracellular Ca^2+^ concentration upon stimulation of 60 mM KCl after pre-incubation of 10 µM CSA or 100 nM nifedipine for 30 min. Values are means ± S.E.M of 4 experiments. *P<0.05 compared with control.

To test the possible role of inhibiting Ca^2+^ influx in CSA-induced relaxation, the arteries were incubated in a Ca^2+^-free depolarizing solution containing 60 mM KCl. The representative trace in Figure 4A shows that the addition of CaCl_2_ into this bathing solution caused concentration-dependent contractions. Thirty-minute treatment with CSA (0.1–10 µM) inhibited CaCl_2_-evoked contraction while nifedipine (L-type calcium channel blocker at 100 nM) was used as positive control (Figure 4B). In addition, CSA inhibited the contraction induced by (−)-Bay K8644 (L-type calcium channel activator at 30 nM) in 15 mM KCl solution (Figure 4C and D) without modifying baseline tension (data not shown). To further confirm the inhibitory effect of CSA on Ca^2+^ influx, Ca^2+^ indicator fluo-4 was used to measure vascular smooth muscle [Ca^2+^]_i_ in isolated renal arteries. Addition of 60 mM KCl caused membrane depolarization and opened voltage-gated Ca^2+^ channel to stimulate Ca^2+^ rise while 30-min pre-incubation of 10 µM CSA reduced this Ca^2+^ rise (Figure 5 A and B). Nifedipine (100 nM) was again used as positive control.

### Roles of Inhibition of Rho Kinase and Protein Kinase C-dependent Mechanisms

**Figure 6:**
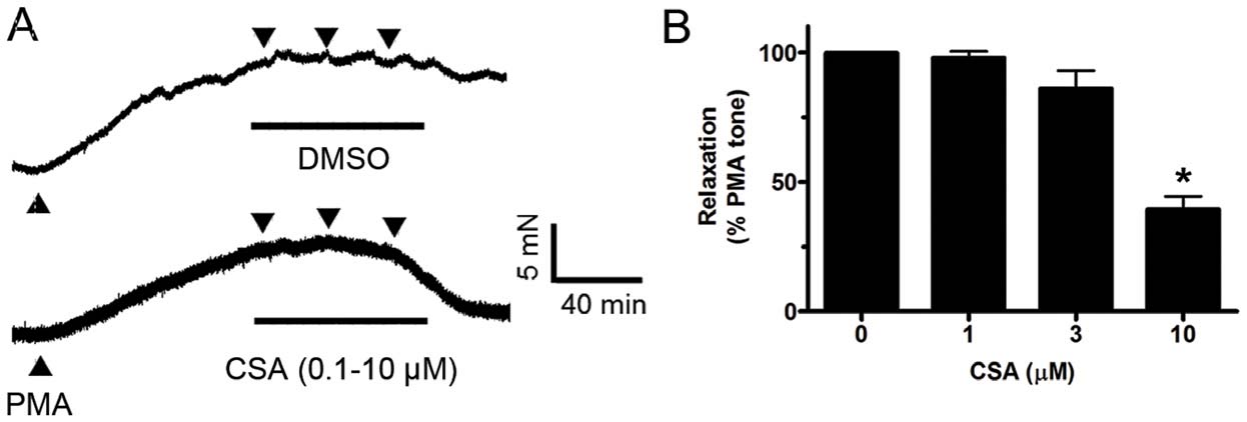
Involvement of PKC and Rho kinase-MLC pathways in CSA-induced relaxation. Representative traces (A) and summarized graph (B) showing relaxation effect of CSA on 10 µM phorbol 12-myristate 13-acetate (PMA)-evoked contraction in Ca^2+^-free Krebs solution containing 100 µM EGTA. Values are means ± S.E.M of 5–6 experiments. *P<0.05 compared with control.

CSA partly suppressed the sustained contraction evoked by phorbol 12-myristate 13-acetate (PMA, protein kinase C activator at 10 µM) in a Ca^2+^-free Krebs solution (Figure 6A and B).

**Figure 7:**
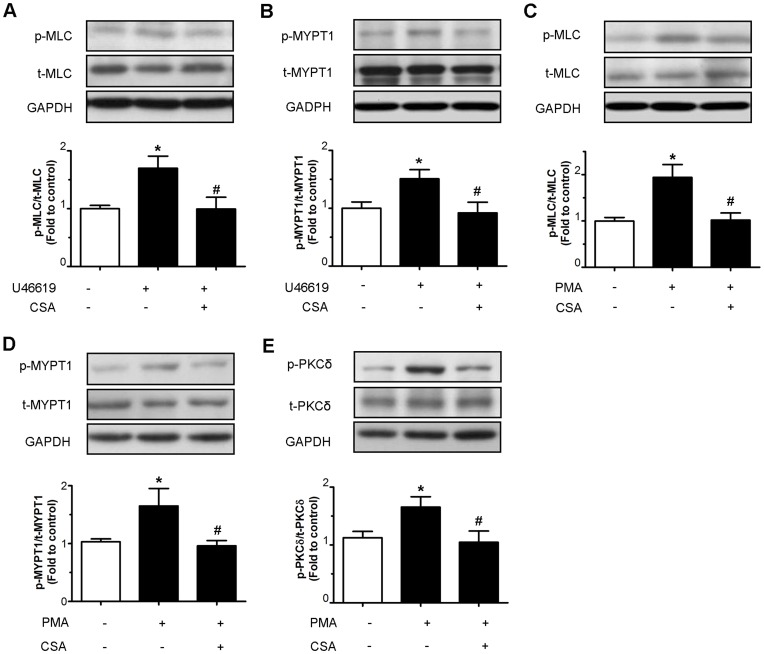
Effect of CSA on phosphorylation of MLC, MYPT1, and PKC. Effect of 10 µM CSA on (A and B) U46619- or (C, D and E) PMA-induced phosphorylation of MLC at Ser19 (p-MLC), MYPT1 at Thr853 (p-MYPT1) and PKCδ at Thr505 (p-PKCδ) as compared to their total levels (t-MLC, t-MYPT1 and t-PKCδ). The lower bands in the t-MYPT1 blot in (B) were unknown proteins probably non-specifically probed by the primary antibodies. Values are means ± S.E.M of 8 experiments. *P<0.05 compared with control and #p<0.05 compared with U46619 or PMA.

Treatment of arteries with 30 nM U46619 for 30 min increased the phosphorylation of MLC at Ser19 (Figure 7A) and MYPT1 at Thr853 (Figure 7B), which were reversed by co-treatment of 10 µM CSA. Furthermore, after treating the arteries with 10 µM PMA in a Ca^2+^-free solution for 60 min, the levels of phosphorylation of MLC at Ser19 (Figure 7C), MYPT1 at Thr853 (Figure 7D) and PKCδ at Thr505 (Figure 7E) were all elevated as compared to the control. Co-treatment with 10 µM CSA reversed the phosphorylation of these signaling molecules (Figure 7).

## Discussion

The present study examined the vascular reactivity of cajaninstilbene acid in rat renal arteries and provided novel findings regarding its pharmacological properties. CSA produced endothelium-independent relaxation (1) partly through antagonism of Ca^2+^ influx via nifedipine-sensitive Ca^2+^ channel and (2) partly through inhibition of Rho kinase and PKC-dependent contractile mechanisms in vascular smooth muscle cells.

Previous studies showed that CSA possesses an estrogen-like activity on osteoblast and osteoclast [14] or ovariectomy-induced bone loss in rats [11]. The present results, however, do not show a gender difference as CSA-induced relaxations were similar in arteries from both male and female rats. In addition, treatment with ICI-182780, a classic estrogen receptor antagonist, did not affect the relaxant effect of CSA.

The endothelium regulates vascular tone, while hypertension and atherosclerosis are associated with the impaired endothelial function; the latter is usually caused by disturbed balance in endothelium-derived relaxing and contracting factors [15]. CSA-induced relaxations were independent of the presence of the intact endothelium nor affected by L-NAME, indomethacin or in arteries contracted by 60 mM KCl, suggesting negligible roles for endothelium-derived relaxing factors. CSA relaxed renal arteries constricted by U46619, serotonin and phenylephrine with similar potency and propranolol was without effect on the CSA relaxation, thus ruling out the interaction of CSA with receptors. It is probable that CSA directly acts on vascular smooth muscle to cause relaxation.

CSA-induced relaxations were not affected by elevated KCl nor by individual blockers for various types of K^+^ channels, thus discounting the involvement of K^+^ channel. Elevated KCl is known to activate voltage-gated Ca^2+^ channels via membrane depolarization in VSMCs. Therefore, high KCl-induced contraction was reduced by CSA, suggesting that CSA is likely to interfere with Ca^2+^ influx via Ca^2+^ channels. This notion was further supported by the following three observations. First, CSA progressively inhibited Ca^2+^-triggered contraction in a Ca^2+^-free, 60 mM KCl-containing Krebs solution. Second, CSA also concentration-dependently reduced contractions evoked by the Ca^2+^ channel activator (−)-Bay K8644 with similar potency as in KCl-evoked contraction. Lastly, CSA prevented the rise of [Ca^2+^]_i_ in smooth muscle cells *in situ* of renal arteries upon the addition of 60 mM KCl.

The present results also suggest that in addition to calcium antagonism other cellular mechanisms may contribute to CSA-induced relaxations as CSA is more effective in relaxing arteries contracted by receptor agonists than by elevated KCl. Constrictive agonists used in this study can activate MLCK, PKC and Rho kinase; the latter two are independent of intracellular Ca^2+^ rise [16], [17], [18]. Both PKC and Rho kinase are involved in the development of hypertension, cerebral and coronary vasospasm, ischemia/reperfusion injury and atherosclerosis [19], [20], [21]. Hence, they can become promising therapeutic targets for the treatment of cardiovascular events. The present results show that CSA partly decreased contraction triggered by the exogenous PKC activator PMA in a Ca^2+^-free Krebs solution, suggesting that PKC inhibition may be involved. The downstream targets of both calcium-dependent and -independent mechanisms mediating vascular smooth muscle contraction are phosphorylation of myosin light chain (MLC) and myosin phosphatase target subunit 1 (MYPT1) [18]. Phosphorylation of MYPT1 decreases its activity to dephosphorylate MLC; and subsequently leading to sustained contraction induced by phosphorylation of MLC [22]. PMA was shown to stimulate phosphorylation of MYPT1 at Thr853 [23], [24] and MCL at Ser19 [25]. U46619 is known to trigger Rho kinase and then phosphorylate MLC and MYPT1 while PMA is the PKC activator. The present study shows that CSA reduced U46619- and PMA-induced phosphorylation of MYPT1 and MLC and phosphorylation of PKCδ. Taken together with the functional results, inhibition of PKC and Rho kinase signaling pathways with reduced phosphorylation of MYPT1 and MLC is likely to account for the part of CSA-induced vasorelaxation.

In summary, the present study provides novel evidence showing that CSA relaxes renal arteries *in vitro* likely through both antagonism of calcium entry via nifedipine-sensitive Ca^2+^ channel and inhibition of cellular pathways in association with PKC and Rho/Rho kinase. The present results indicate that CSA and CSA-containing herbs can be of potential benefits in reducing the elevated VSMC tension which is implicated in cardiovascular pathogenesis although such benefit needs in-depth investigation in animal models of hypertension and other vascular pathologies.

## Materials and Methods

### Artery Rings Preparation

The investigation conforms to the Guidelines for the Care and Use of laboratory animals published by the National Institutes of Health. Both male and female Sprague-Dawley rats (250∼300 g) were killed by cervical dislocation. The kidneys were removed and dissected in ice-cold oxygenated Krebs solution. Krebs solution contained the following composition (in mM): 119 NaCl, 4.7 KCl, 1 MgCl_2_, 2.5 CaCl_2_, 25 NaHCO_3_, 1.2 KH_2_PO_4_ and 11 D-glucose.

### Isometric Force Measurement

Rings (∼2 mm in length) of renal arteries were isolated from adhering connective tissues. Each segment was mounted in a Multi Myograph System (Danish Myo Technology A/S, Denmark), bathed in Krebs solution bubbled with 95% O_2_–5% CO_2_ and maintained at 37°C at pH ∼7.4 as described previously [26]. Renal arterial rings were set to an optimal tension of 2 mN and stabilized for 90 min. The rings were then contracted by 0.5 µM phenylephrine and challenged with 3 µM acetylcholine to confirm the integrity of the endothelium. In some protocols, the endothelium was mechanically removed via rubbing the internal surface of arteries with a stainless steel wire and verified by the lack of relaxation in response to 3 µM acetylcholine.

Phenylephrine (0.5 µM), U46619 (100 nM), serotonin (1 µM) and KCl (60 mM) were used to induce steady contraction tone in endothelium-intact rings, concentration-response curves were subsequently studied by cumulative addition of cajaninstilbene acid (CSA, 0.1–10 µM). The time-matched vehicle (DMSO) control protocol was also performed.

In the second set of experiments, CSA-induced relaxation was obtained in endothelium-intact and endothelium-denuded rings. Some rings with intact endothelium were exposed for 30 min to L-NAME (nitric oxide synthase inhibitor, 100 µM) [27], indomethacin (non-selective cyclooxygenase inhibitor, 3 µM) or ICI-182780 (estrogen receptor antagonist, 10 µM) [27]; while some rings without endothelium were pretreated for 30 min to TEA^+^ (3 mM), BaCl_2_ (10 µM), glibenclamide (10 µM), 4-AP (1 mM) or propranolol (1 µM).

To determine the effect of CSA involved in Ca^2+^ influx through L-type Ca^2+^ channels, CaCl_2_ (0.01–5 mM) was added in Ca^2+^-free, 60 mM KCl solution containing 100 µM EGTA after pre-incubation of CSA (0.1, 1, 3, or 10 µM) or nifedipine (L-type calcium channel blocker, 100 nM) for 30 min. In addition, some rings were pre-contracted by (−)-Bay K8644 (calcium channel opener, 30 nM) in 15 mM KCl Krebs solution [28], [29] to study CSA-induced relaxation.

Finally, the calcium-independent mechanisms were studied in endothelium-denuded rings. Each ring was contracted by phorbol 12-myristate 13-acetate (PMA at 10 µM) in a Ca^2+^-free Krebs solution containing 100 µM EGTA and then relaxed by CSA.

### Calcium Fluorescent Imaging in Renal Artery

Renal arteries were dissected free from connective tissue and cut open longitudinally. The vascular strips were incubated with 5 µM calcium indicator fluo-4 AM (Molecular Probes) in Krebs solution for 1 h at 37°C as described before [30]. Some strips were co-treated with 10 µM CSA or 100 nM nifedipine for 30 min. The strips were then bathed in organ chambers filled with Krebs solution bubbled by 95% O_2_ and 5% CO_2_ and 10 mM BDM (2,3-butanedione monoxime, myosin inhibitor, Sigma) at 37°C. Using an Olympus Fluoview FV1000 laser scanning confocal system (Olympus), fluorescence was measured continuously every 20 s (excitation: 495 nm and emission: 505–525 nm). The fluorescence intensity at a certain time point (F_1_) was compared to that at the starting point of image recording (F_0_) to show the changes of intracellular Ca^2+^ concentration [Ca^2+^]_i_ upon the addition of 60 mM KCl.

### Western Blotting

After incubation with 10 µM PMA in Ca^2+^-free Krebs solution containing 100 µM EGTA with or without 10 µM CSA for 60 min or incubation with 30 nM U46619 with or without 10 µM CSA for 30 min in Krebs solution, renal arteries were frozen in liquid nitrogen and homogenized in ice-cold RIPA lysis buffer containing 1 µg/ml leupeptin, 5 µg/ml aprotonin, 100 µg/ml PMSF, 1 mM sodium orthovanadate, 1 mM EGTA, 1 mM EDTA, 1 mM NaF, and 2 mg/ml β-glycerolphosphate as described previously [31]. The lysates were centrifuged at 20,000 g for 20 min to obtain supernatants. The protein sample was quantified by the Lowry method (BioRad). Protein sample (20 µg) was electrophoresed through the SDS-polyacrylamide gel and transferred to an immobilon-P polyvinylidene difluoride membrane (Millipore). The membranes were blocked with 1% BSA and incubated with primary antibodies against phosphorylated MLC at Ser19 (p-MLC), total MLC (t-MLC) (Sigma), phosphorylated PKCδ at Thr505 (p-PKCδ), total PKCδ (t-PKCδ), phosphorylated MYPT1 at Thr853 (p-MYPT1) (Cell Signalling), total MYPT1 (t-MYPT1) (Covance), and GAPDH (Ambion) at 4°C overnight, followed by horseradish peroxidase-conjugated secondary antibodies (DakoCytomation) and developed with an enhanced chemiluminescence detection system (ECL reagents; Amersham Pharmacia Biotech). Densitometry was performed with a documentation program (Flurochem) and analyzed with QuantityOne (Biorad).

### Chemicals

Cajaninstilbene acid (purity≥99%) was isolated from *Cajamus cajan* (L.) Millsp. Phenylephrine, acetylcholine, serotonin, L-NAME, indomethacin, TEA^+^, ICI-182780, glibenclamide, BaCl_2_, 4-AP, propranolol, nifedipine, (−)-Bay K8644, and PMA were from Sigma and U46619 was from Cayman. Indomethacin, ICI-182780, glibenclamide, nifedipine, (−)-BayK 8644, PMA, U46619 and CSA were dissolved in DMSO and others in double-distilled water.

### Statistical Analysis

Data are means±SEM of n rats. pD_2_ refers to the negative logarithm of the dilator concentration that caused half of the maximal relaxation and E_max_% refers to the maximum relaxation. Concentration-response curves were analyzed via GraphPad software (Version 4.03) and statistical significance was compared by two-tailed Student’s t-test or one-way analysis of variance followed by Newman-Keuls test. Values of p<0.05 was considered statistically significant.
